# Nutrient release and yield comparison between customized organic and conventional fertilizers in hydroponic lettuce production

**DOI:** 10.3389/fpls.2026.1785126

**Published:** 2026-02-26

**Authors:** Sangrak Son, Lori Hoagland, Amanda Deering, Jin Wei-Kocsis, Krishna Nemali

**Affiliations:** 1 Department of Horticulture and Landscape Architecture, Purdue University, West Lafayette, IN, United States; 2 Department of Food Science, Purdue University, West Lafayette, IN, United States; 3 Polytechnic Institute, Purdue University, West Lafayette, IN, United States

**Keywords:** electrical conductivity, growth rate, leaf color, plant tissue analysis, residual fertilizer

## Abstract

Hydroponic operations can receive organic labelling in the US. Organic labelling has the potential to increase profits in hydroponic production as organic products are sold at a premium price. Nutrient uptake by plants follows a sigmoidal pattern, with approximately a third of the nutrients taken during the lag (slow growth) phase and the remaining two-thirds of nutrients taken by plants during the log (rapid growth) phase. Nutrient release from fertilizers should match nutrient demand from plants for optimal crop growth. There is limited information about nutrient release from organic fertilizers, their uptake by plants, and the overall effect on crop yield in hydroponic production. Eight lettuce cultivars, belonging to four morphological groups and two leaf colors, were grown in containers filled with peat-based substrate containing either customized organic or conventional fertilizer mixes with a similar quantity of different nutrient elements. Reverse osmosis water was used to sub-irrigate plants in a recycling hydroponic system. Crop yield and nutrient release differences between organic and conventional treatments were quantified in the study. Results indicated that the average fresh shoot biomass of lettuce cultivars was approximately 36% lower in the organic than in the conventional fertilizer treatment. However, plants in both treatments visibly appeared to be of similar quality. The electrical conductivity of the substrate (i.e., total soluble nutrients in the root zone) was significantly lower during the exponential (or rapid) growth stage (0.80 vs 1.23 dS.m^-1^), indicating lower nutrient availability for plants in organic than conventional treatment. Further, biomass reduction in organic compared to conventional treatment was larger for fast-growing varieties and green than red leaf cultivars, suggesting greater yield losses in cultivars with higher nutrient demand. Interestingly, a high dose of customized organic fertilizer (approximately twice) incorporated into the substrate resulted in a comparable yield (48.9 g·plant^-^¹) with that of the conventional fertilizer treatment (45.8 g·plant^-^¹). However, a significant quantity of residual nutrients was found in the substrate of the customized high-dose organic fertilizer treatment. A high dose of customized organic mix plus a strategy to reuse the substrate can potentially increase yield and nutrient use in hydroponic production.

## 1 Introduction

Hydroponics involves growing plants solely in a nutrient solution or in soilless substrates (Maher et al., 2007). This method of farming has several benefits, including reduced water use compared to field-based farming (Naresh et al., 2024; Alizaeh et al., 2025), year-round crop production regardless of seasonality (Barbosa et al., 2015; Lee and Lee, 2015; Khatri et al., 2024), and optimized nutrient delivery for rapid growth and shorter crop production cycles (Barbosa et al., 2015; Majid et al., 2021). Moreover, crop productivity (e.g., lettuce) is comparable between hydroponic and field-based farming systems (Lei and Engeseth, 2021). However, economic sustainability is a major challenge in hydroponic farming due to high initial investment and operational costs, leading to low profit margins (Naresh et al., 2024; Souza et al., 2019; Velazquez-Gonzalez et al., 2022).

Customer interest in organic products is rapidly increasing globally due to their benefits to personal wellness and the environment (Michaelidou and Hassan, 2008; Gundala and Singh, 2021; Nafees et al., 2022). Lettuce is one of the major organically grown crops in the US. In 2024, the wholesale value of organic lettuce sold in the US was $382 million (Organic Produce Network, 2024). Generally, organic crops are sold at higher retail prices than conventionally-grown crops (McBride et al., 2015). For example, a meta-analysis of various crops indicated that the price of organically grown produce was 29-32% higher than that of conventional produce in the US (Crowder and Reganold, 2015). Interestingly, hydroponic operations in the US can receive organic certification if they comply with the regulations set by the United States Department of Agriculture (USDA). Accordingly, hydroponic operations must use organic-certified materials, including organic substrates, organic-certified seeds or transplants, and approved organic fertilizers and pesticides (Maher et al., 2007; Park and Williams, 2024), to receive organic certification. Given that organic products are sold at a premium, it may be possible to increase revenue from hydroponic production by obtaining organic certification for hydroponically grown crops. This may potentially increase profits and improve the economic sustainability in hydroponics industry.

Nutrient uptake by plants follows a sigmoidal path, with approximately a third of the nutrients taken during the lag (slow growth) phase and the remaining two-thirds taken during the log (rapid growth) phase (Solis-Toapanta et al., 2020). A key aspect of hydroponic production is the presence of highly soluble nutrients from conventional (synthetic) fertilizers in the root zone to support rapid plant growth. To match the nutrient demand, nutrient release (e.g., controlled-release conventional fertilizers) also follows a similar trend (Chang-wen et al., 2006). Given this, the economic benefit of using organic practices is contingent on matching the nutrient release from organic fertilizers with the nutrient demand from plants. If there is a mismatch between nutrient release from organic fertilizers and nutrient demand from plants, expected high crop yields may not be possible in hydroponic production.

There is limited information related to nutrient release from organic fertilizers in hydroponic systems. Although many studies compared organic and conventional production in field-grown crops, there are limited studies that quantified crop yield responses with organic fertilizers in hydroponic systems. The objectives of our research were to (i) compare differences in nutrient release and their uptake by plants provided with customized organic and conventional fertilizers containing similar amounts of nutrient elements, (ii) quantify yield differences in hydroponically-grown lettuce cultivars supplied with the customized organic and conventional fertilizer mixes, and (iii) develop organic fertilizer management strategies that result in comparable lettuce yield with that of conventional fertilizers in hydroponic production.

## 2 Materials and methods

### 2.1 Experiments

The study comprised of two experiments to address the objectives. Experiment I quantified yield differences and compared nutrient release and uptake patterns with customized organic and conventional fertilizers, while experiment II focused on organic fertilizer management strategies. There were two trials in experiment II. Both trials were similar except that the substrate from trial 1 was reused in trial 2 without any addition of fertilizers. Each trial in experiments I and II was for 20 and 30 days, respectively. Both experiments were conducted in a glass greenhouse located at Purdue University (West Lafayette, IN, USA).

### 2.2 Seeds and sowing

Organic seeds of eight lettuce cultivars, representing four morphological groups (leaf, romaine, butterhead, and oak leaf) and two leaf colors (green and red), were procured (Johnny’s Selected Seeds, Winslow, ME) for our study (**Table 1**). Seeds were sown in a peat-based germination medium (BM2, Berger, QC, Canada) filled in 72-cell seed trays (54 cm × 27 cm × 3 cm; Greenhouse Megastore, Danville, IL). Ten days after sowing, seedlings were transplanted into square plastic pots (9.3 cm × 9.3 cm × 7.9 cm; Greenhouse Megastore, Danville, IL) filled with a custom prepared soilless substrate using organically-approved sphagnum peat moss (Sun Gro Horticulture, Agawam, MA, USA) and vermiculite (Old Castle Lawn and Garden, Atlanta, GA, USA) in a 4:1 (peat moss: vermiculite, v/v) ratio.

**Table 1:** Different cultivars used in the study with their group name, leaf color, and growth rates.

Cultivar	Group	Leaf color	Growth rate^1^
Waldmann’s dark green	Leaf	Green	Fast
Red sails	Leaf	Red	Fast
Buttercrunch	Butterhead	Green	Medium
Alkindus	Butterhead	Red	Medium
Dragoon	Romaine	Green	Medium
Breen	Romaine	Red	Medium
Bauer	Oak leaf	Green	Medium
Red salad bowl	Oak leaf	Red	Fast

^1^Manufacturer provided information.

### 2.3 Fertilizer preparation

All fertilizers were directly incorporated into the substrate before potting, except in trial 2 of experiment II, where no fertilizers were added (see treatment description below). Two types of fertilizers, i.e., conventional and customized organic mixes, were custom-prepared (**Table 2**). A slow-release fertilizer (Osmocote) was combined with gypsum to prepare the conventional fertilizer mix (hereafter ‘conventional mix’). Different OMRI (Organic Materials Review Institute) certified organic fertilizers were combined to prepare the customized organic fertilizer mix (hereafter ‘organic mix’). Both conventional and organic mixes were formulated to ensure that plants receive most of the essential macronutrients and micronutrients. Because the concentration of nutrients was generally less in the organic components, more quantity of organic mix (4.6 g·pot^-1^) was added to the substrate than conventional mix (2.0 g·pot^-1^) to balance the supply of different nutrient elements to plants (**Table 2**).

**Table 2:** List of fertilizers used in preparing customized conventional and organic mixes, and their approximate weights in each pot.

Type	Fertilizer components	Weight/pot (mg)	Manufacturer
Conventional	Osmocote 15-9-12	1750	Everris NA Inc.
	Gypsum	250	MK Minerals Inc.
	Total	2000	
Organic	7-5-7	3333	California Organic Fertilizer Inc.
	4-3-2	830	D. Stutzman Farms
	Epsom Salt	250	Lawn and Garden Products Inc
	Ferrous Sulfate	83	Brandt Consolidated Inc
	Manganese Sulfate	8	Brandt Consolidated Inc
	Fertibor	42	U.S. Borax Inc.
	Total	4546	

Both conventional and organic mixes had similar amounts of several nutrient elements (**Table 3**). However, the organic mix contained a slightly lower amount of P_2_O_5_ and S and did not contain Mo, Cu, and Zn, compared to the conventional mix. On the other hand, the organic mix contained higher amounts of Fe, Mn, and B than the conventional mix.

**Table 3:** Quantity of different elements in the conventional and organic fertilizer mixes.

Fertilizer	N	P_2_O_5_	K_2_O	Ca	Mg	S	Fe	Mn	Mo	B	Cu	Zn
mg												
Conventional	263	158	210	53	23	141	8.1	1.1	0.4	0.4	0.9	0.9
Organic	267	83	208	58	24	43	16.7	1.3	–	6.3	–	–

### 2.4 Hydroponic system

A closed-loop recirculating hydroponic system was used to grow plants. The hydroponic system utilized trays (121.9 cm × 30.5 cm × 8.9 cm; Botanicare, Vancouver, WA), which were placed on greenhouse benches (762 cm × 150 cm × 110 cm) and connected to reservoirs (76 L; Active Aqua Premium, Petaluma, CA) placed on the ground. Reverse osmosis (RO) water was filled (approximately 40 L) in each reservoir for irrigation. The reservoirs were thoroughly washed before the experiment to remove any traces of fertilizer from previous trials. In addition, no fertilizer was added to the RO water in the reservoirs during experiments. Plants absorbed fertilizer present only in the substrate. The inlet of the tray was connected to a submersible pump (530 L·hr^-1^; Total Pond, West Palm Beach, FL) located inside the reservoir using a vinyl tubing (1.6 cm ID, Crop King Inc., Lodi, OH). The pump was operated for 15 minutes each day for sub-irrigation (i.e., uptake of water by the substrate through the holes at the bottom of the pot). The flow of water to the trays was adjusted to be uniform among different units using valves (Green Back in-line valve; Botanicare) attached to the inlet tubing. A short vertical pipe (5 cm) was inserted into the outlet hole to control the level of water that accumulates in the tray before draining back to the reservoir. This was necessary to create the pressure head needed for subirrigation. After the pump was turned off, any remaining water in the tray drained back into the reservoir through the inlet tubing.

### 2.5 Greenhouse environment

Environmental conditions in the greenhouse, including air temperature, relative humidity, and light intensity, were continuously monitored during both experiments. Air temperature was measured using thermistors (ST-100; Apogee Instruments, Logan, UT) and light intensity was recorded using quantum sensors (SQ-500; Apogee Instruments, Logan, UT). The temperature and quantum sensors were connected to a datalogger (CR1000; Campbell Scientific, Logan, UT) for continuous data collection. Relative humidity was measured by wet/dry bulb thermistors of a psychrometer (Priva Controller, Camarillo, CA). The environmental conditions inside the greenhouse during both experiments included average air temperature of 22.1 ± 2.49 °C, average relative humidity of 53 ± 11.2%, and average daily light integral (a measure of total amount of light received in a day) of 16.4 ± 4.69 mol·m^-2^·d^-1^.

### 2.6 Treatments

Treatments in experiment I consisted of two fertilizer types and eight lettuce cultivars. The two fertilizer treatments were conventional (2 g·pot^-1^) and organic (4.6 g·pot^-1^) mixes (**Table 2**). Both fertilizer treatments were incorporated into the substrate before the start of experiments. The eight lettuce cultivars (**Table 1**) and were grown in each fertilizer treatment. Experiment II consisted of two separate trials. Each trial had two fertilizer treatments including conventional (2 g·pot^-1^) and high-dose organic (8 g·pot^-1^) mix treatments. The composition of conventional and organic mixes was similar to those in experiment I, except that a higher quantity of organic mix was incorporated in the high-dose organic treatment. Two leaf lettuce cultivars (‘Waldmann’s dark green’ and ‘Red sails’) were grown in each of the fertilizer treatments. These cultivars were selected based on their fast growth observed in experiment I, which will likely increase nutrient demand from the fertilizers. The conventional and high-dose organic mix treatments were incorporated into the substrate at the start of the first trial. After completing the first trial, the plant roots and debris were removed from the substrate in each pot. The cleaned substrate was refilled into the pots by maintaining the original treatment integrity. The substrate was reused to grow plants with residual fertilizer (i.e., without addition of any fertilizer) in the second trial.

### 2.7 Measurements

In experiment I, measurements involved fresh weight (FW, a measure of economic yield in lettuce) and dry weight (DW) of plants in all treatments at the harvest stage. The substrate electrical conductivity (EC_sub_) in all treatments was measured at three time points, including 1^st^, 10^th,^ and 20^th^ day after transplanting (DAT). In addition, the elemental composition of plant (shoot) tissue was measured at the harvest stage for ‘Red sails’ cultivar in both fertilizer treatments. This cultivar was chosen due to its well-known rapid growth rate in hydroponic systems. In experiment II, FW and DW were measured in all treatments and both trials at the harvest stage. In addition, the leaf tissue nitrogen content (N_leaf_) was measured in trial 1 and EC_sub_ was measured in trial 2 at the harvest stage in all treatments.

The FW was measured by harvesting plants at the ground level and weighing the shoots immediately using a precision scale (ML 1602T/00; Mettler-Toledo, LLC, Columbus, OH). The DW was measured by placing the shoot material in a bag and drying it in a forced air oven maintained at 70 °C for a week, and weighing the dried material using the scale. The EC_sub_ was measured using a 5TE dielectric sensor (Metergroup, Pullman, WA, USA) inserted into the substrate an hour after a sub-irrigation event. The elemental composition in the plant tissue was measured using dried shoot material at a commercial laboratory (A & L Laboratories, Fort Wayne, IN). The sufficiency and deficiency ranges of nutrient elements in the plant tissue of hydroponically grown leaf lettuce was determined using the Plant Analysis Handbook (Bryson et al., 2014). The N_Leaf_ was measured using dried shoot materials. A nitrogen analyzer (Rapid N Exceed; Elementar Americans Inc, Ronkonkoma, NY) based on the ‘Dumas’ combustion method was used for estimating nitrogen content in the plant material. The analyzer combusts the sample and converts it into gaseous products. After reduction and purification, nitrogen was quantified as N_2_ by a thermal conductivity detector. Later, quantified N_2_ was converted to nitrogen content in the sample using a manufacturer provided calibration.

### 2.8 Statistical analyses

Experiments I and II were laid out in a split-plot design, with fertilizer (two levels) as the main plot factor and cultivar (eight or two cultivars) as the subplot factor. The main plot was replicated four times. Data were analyzed for main and interactive effects of fertilizer, cultivar, and measurement time using a general linear model (Proc GLM) of statistical analysis software (SAS, version 9.4, SAS Institute, Cary, NC, USA). Appropriate error terms for main and sub-plot treatments were used in the analyses. Repeated measures analysis was used to test the interactions associated with measurements performed on plants over time. The least-square means were compared using Tukey’s honestly significant difference (HSD) test at a significance level of *P* ≤ 0.05. Linear regressions were tested for the significance of the slope parameter using the regression procedure of SAS. All graphs and regression plots were made using SigmaPlot software (version 14.0, Systat Software Inc., San Jose, CA).

## 3 Results

### 3.1 Experiment I

#### 3.1.1 Effect of conventional and organic mixes on shoot biomass

**Figure 1:**
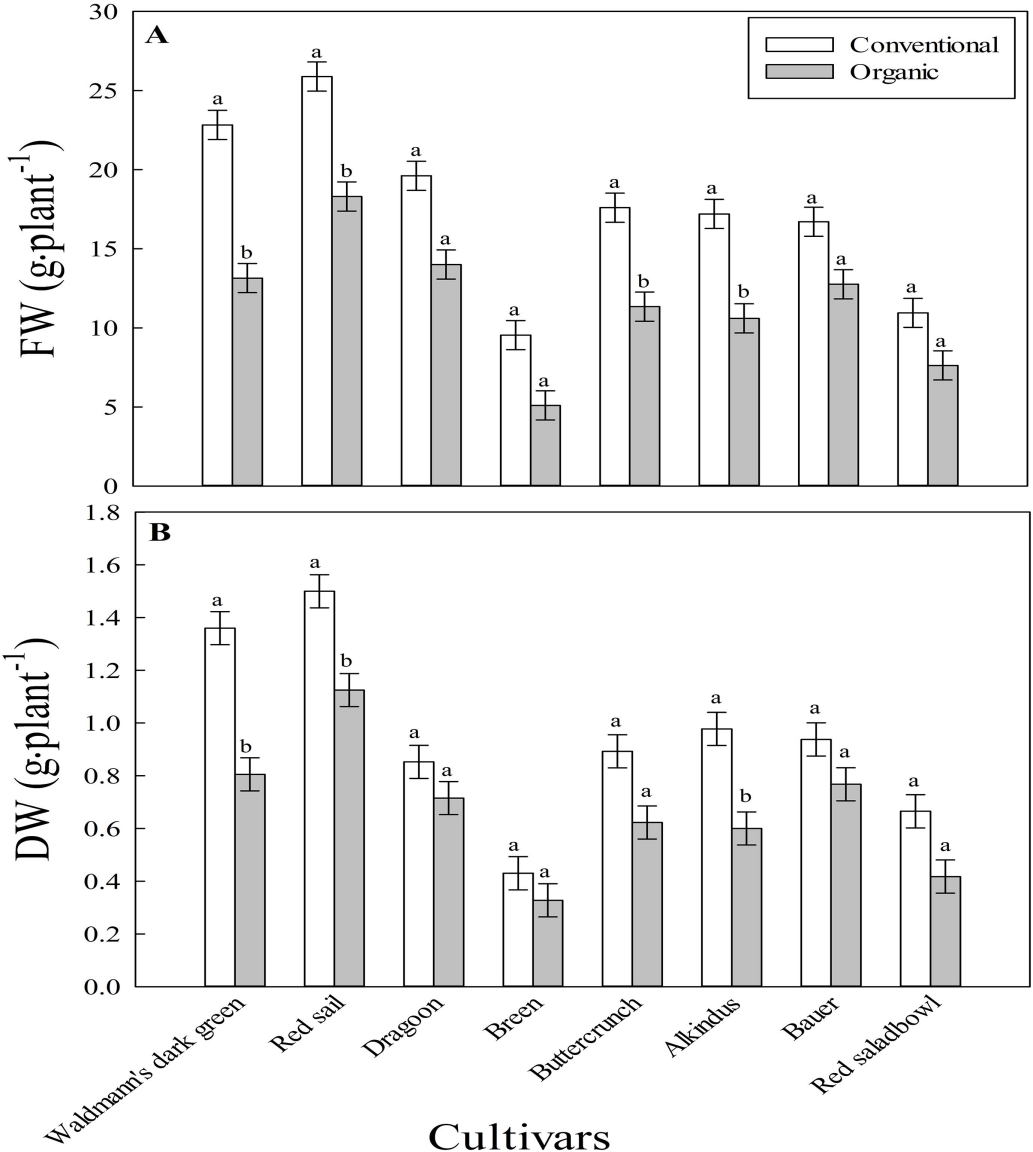
Interactive effect of fertilizer type (conventional and organic mixes) and cultivar (eight lettuce cultivars) on **(A)**. fresh weight (FW; g·plant^-1^) and **(B)**. dry weight (DW; g·plant^-1^) in experiment I. Each bar represents the average of four replications (n=4). The least square means with different letters are statistically different (*P* < 0.05) between the two fertilizer treatments within each cultivar. Error bars indicate the standard error of the mean.

There was a significant interaction between fertilizer type and cultivar for FW and DW (**Figure 1**). This indicates that the shoot biomass response to fertilizer type varied among cultivars. In general, the interactive effects were similar for both FW and DW. Cultivars including ‘Alkindus’, ‘Buttercrunch’, ‘Red sails’, and ‘Waldmann’s dark green’ had significantly higher FW in conventional than organic mix (**Figure 1**). On average, FW was 36.1% higher in the conventional than organic mix among these four cultivars. Other cultivars, including ‘Breen’, ‘Dragoon’, ‘Bauer’, and ‘Red salad bowl’ showed numerically higher FW in the conventional than organic mix without statistically significant differences. The higher yield in the conventional than organic mix was significant in the leaf and butterhead groups but not in the romaine and oakleaf groups of lettuce in our study.

**Figure 2:**
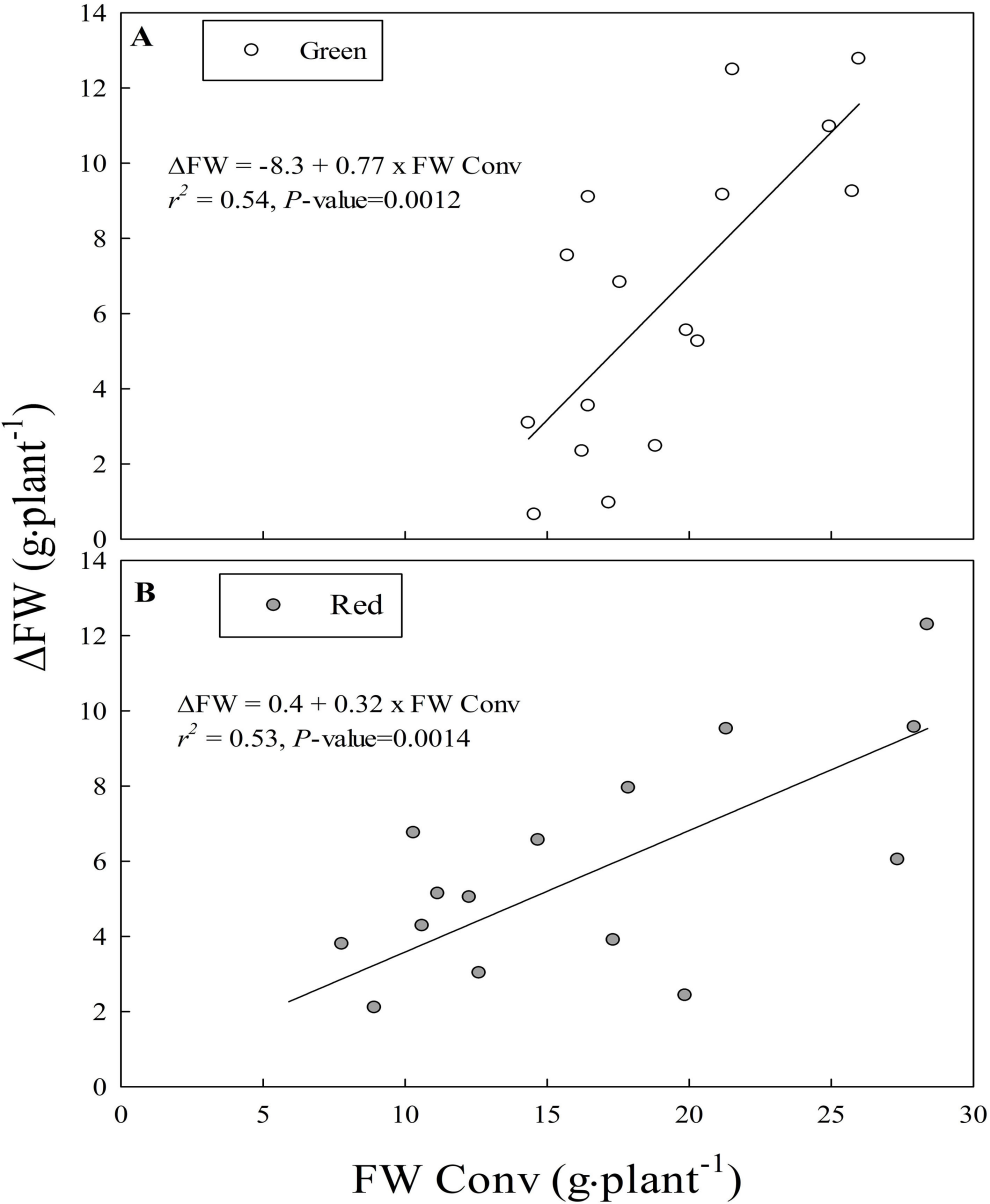
Relationship between yield gap (Δ FW, g·plant^-1^) and fresh weight of cultivar in the conventional treatment (FW Conv, g·plant^-1^) in **(A)**. green leaf cultivars and **(B)**. red leaf cultivars of lettuce in experiment I. Data from four cultivars and four replications (N = 16) were shown for each leaf color. The fitted linear equations are shown as graph inserts.

There was a linear relationship between a cultivar’s ‘yield gap’ (i.e., difference in FW of a cultivar between the conventional and organic treatments) and its FW in the conventional treatment (**Figure 2**). In other words, larger yield gaps were observed in cultivars exhibiting larger size in the conventional treatment. However, the magnitude of yield gap depended on the cultivar’s leaf color with green leaf cultivars showing a larger yield gap (slope of 0.74; **Figure 2A**) than red leaf cultivars (slope of 0.32; **Figure 2B**) with increasing FW in the conventional treatment.

#### 3.1.2 Effect of conventional and organic mixes on substrate electrical conductivity

**Figure 3:**
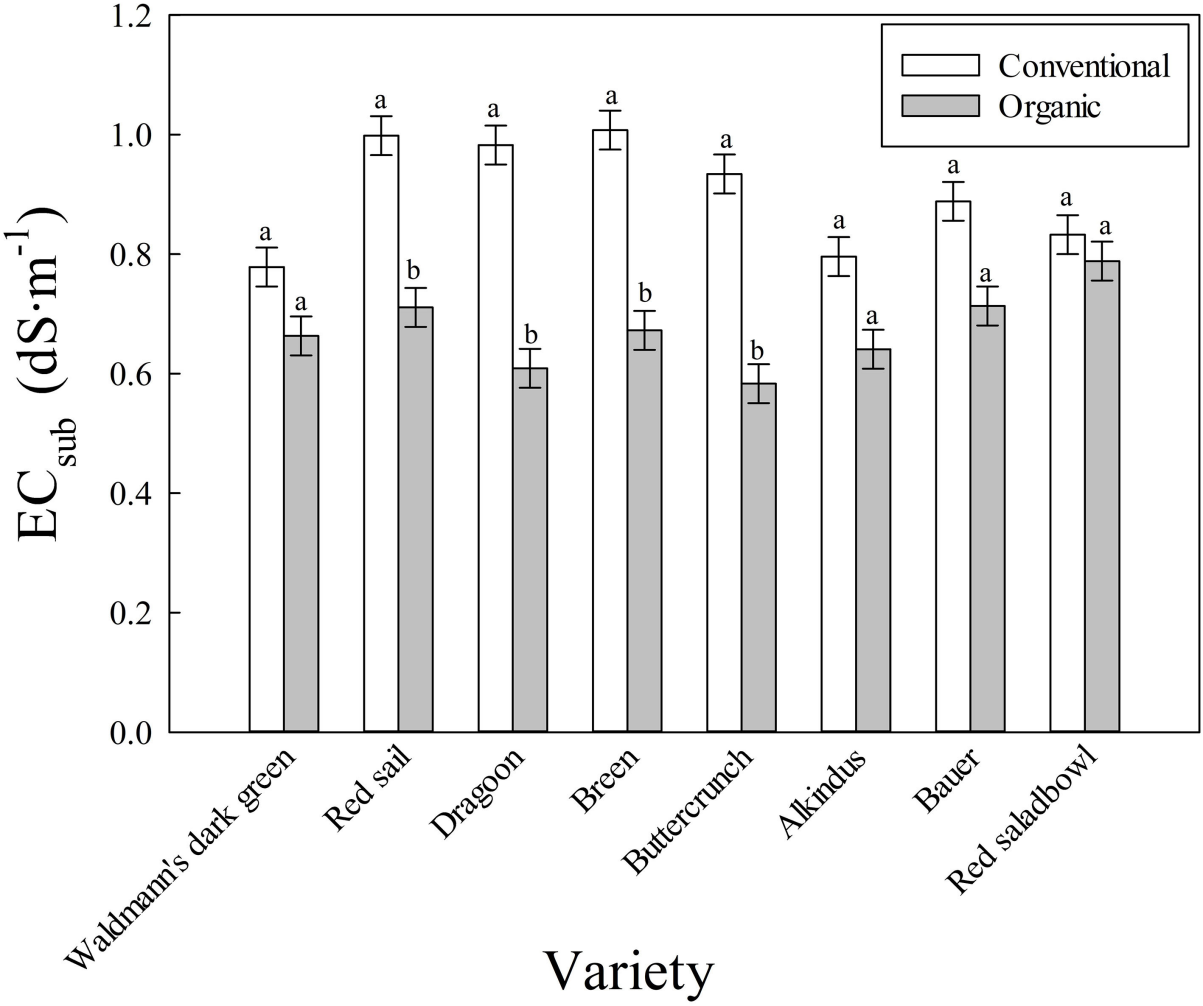
Interactive effect of fertilizer type (conventional and organic mixes) and cultivar (eight lettuce cultivars) on the electrical conductivity of substrate (EC_sub_, dS·m^-^¹) in experiment I. Each bar represents the average of three measurement times and four replications (N = 12). Least square means with different letters are statistically different (*P* < 0.05) between the two fertilizer treatments within each cultivar. Error bars indicate the standard error of the mean.

A significant interaction between fertilizer type and cultivar was observed for EC_sub_ (**Figure 3**). This indicates that the effect of fertilizer treatments on EC_sub_ varied among lettuce cultivars. The average EC_sub_ in the conventional mix was significantly higher than that of the organic mix for ‘Bauer’, ‘Breen’, ‘Buttercrunch’, ‘Dragoon’, and ‘Red sails’ cultivars. In these cultivars, the average EC_sub_ ranged between 0.89 and 1.01 dS·m^-^¹ in the conventional mix, whereas the average EC_sub_ range in the organic mix was between 0.58 and 0.71 dS·m^-^¹. However, average EC_sub_ in ‘Alkindus’, ‘Red salad bowl’, and ‘Waldmann’s dark green’ was not statistically different (but numerically higher) between the conventional and organic mixes (0.78 to 0.83 dS·m^-^¹ in conventional and 0.64 to 0.79 dS·m^-^¹ in organic mix).

**Figure 4:**
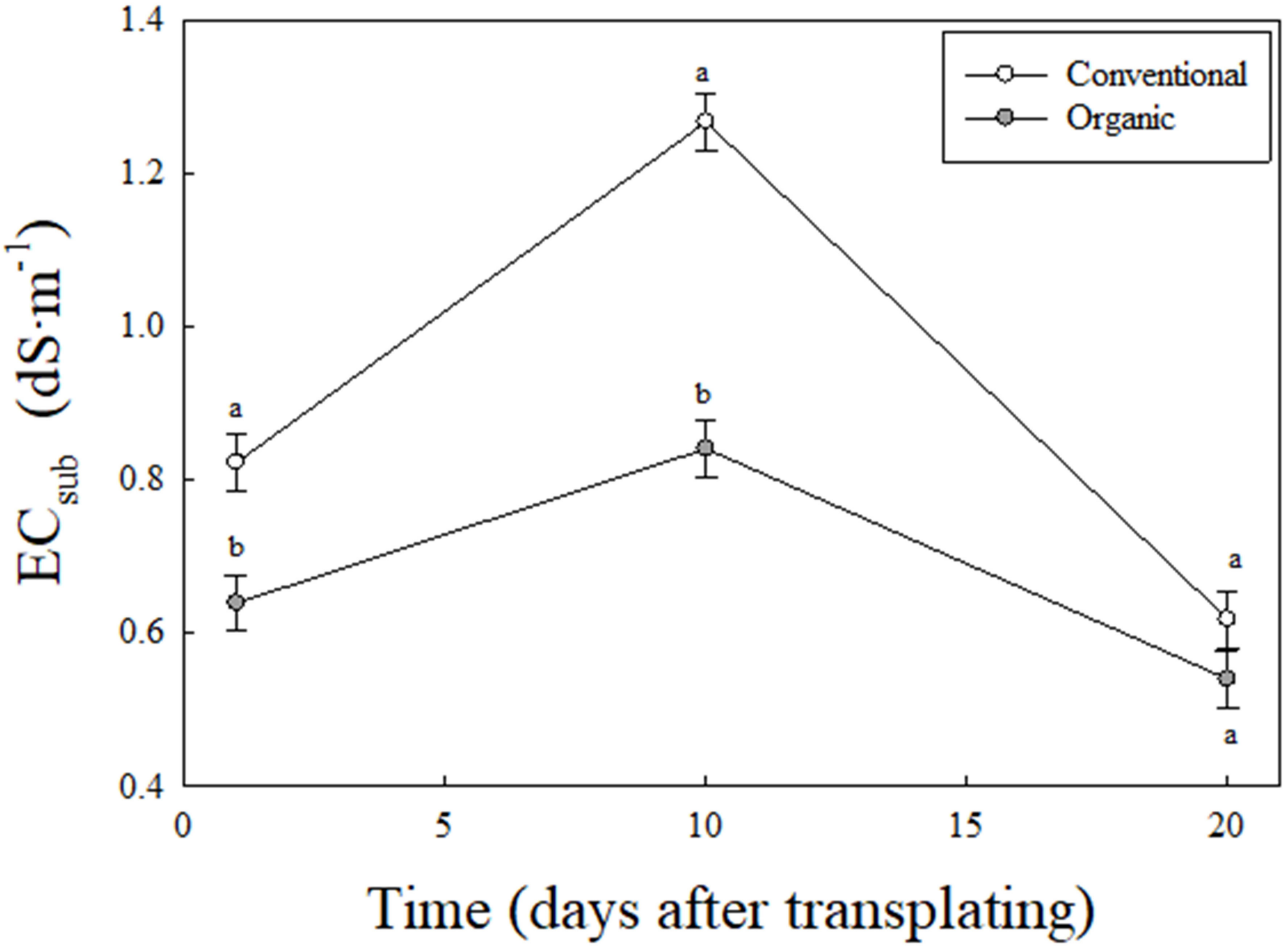
Interactive effect of fertilizer type (conventional and organic mixes) and measurement time (1, 10, 20 DAT) on the electrical conductivity of substrate (EC_sub_, dS·m^-^¹) in experiment I. Each data is an average of eight varieties and four replications (N = 32). Least square means with different letters are statistically different (*P* < 0.05) between the two fertilizer types at any given measurement time. Error bars indicate the standard error of the mean.

In addition, a significant interaction between fertilizer type and measurement time was observed for EC_sub_ (**Figure 4**). This indicates that the effect of fertilizer treatment on EC_sub_ varied by the measurement time. The EC_sub_ in the conventional mix was significantly higher than organic mix at 1 and 10 DAT but not at 20 DAT. In the conventional mix, EC_sub_ sharply increased by approximately 35% from 0.82 to 1.27 dS·m^-^¹ between 1 and 10 DAT, followed by a significant decrease of approximately 51% from 1.27 to 0.62 dS·m^-^¹ between 10 and 20 DAT. In contrast, EC_sub_ in the organic treatment showed a moderate increase of approximately 23% from 0.64 to 0.84 dS·m^-^¹ between 1 and10 DAT, then decreased by approximately 36% from 0.84 to 0.54 dS·m^-^¹ between 10 and 20 DAT.

#### 3.1.3 Effect of conventional and organic fertilizer mixes on elemental composition of plants

A main effect of fertilizer treatment was observed on the elemental composition of plants in ‘Red sails’ cultivar (**Table 4**). Among different elements, N, S, Zn, and Cu were found in significantly higher levels in the conventional than organic mix. Whereas K, Ca, Mn, and B were found in significantly higher levels in the organic than conventional mix. Elements including P, Mg, Fe, and Al were not statistically different between the fertilizer mixes. The N level of plants in the organic mix was sub-optimal (deficient) and lower by 37.6% than that of the conventional mix (**Table 4**). However, the levels of S, Zn, and Cu, despite being significantly lower than conventional mix, were within the sufficiency range in the organic mix. Although the levels of K, Mn, and B were significantly lower than that of organic mix, they were within the sufficiency range in the conventional mix. Supra-optimal level of Mn was observed in both treatments and that of B was observed in the organic mix. There were no toxicity symptoms of these elements in plants in both fertilizer treatments. The level of Ca in the tissue was found to be deficient in both organic and conventional mixes, albeit at a higher level in the organic mix. The levels of P, Fe, and Al were within the sufficiency range in both treatments. Although not statistically different between the two mixes, Mg was in the deficiency range in the organic mix.

**Table 4:** Composition of different elements in plants subjected to conventional and organic mixes in experiment I.

Element	Units	Conventional	Organic	Sufficiency range^1^
N	mg·g^-1^	47.6 (0.21) a	29.7 (0.08) b	37.5 – 56.0
P		8.1 (0.04) a	7.7 (0.03) a	4.5 – 7.7
K		39.8 (0.13) b	66.0 (0.13) a	30.0 – 65.0
Ca		6.2 (0.02) b	8.2 (0.03) a	12.5 – 25.0
Mg		5.6 (0.01) a	3.4 (0.01) a	4.5 – 7.8
Na		0.9 (0.01) b	1.8 (0.01) a	0.1 - 2.4
S		4.5 (0.01) a	3.7 (0.02) b	2.5 - 3.5
Zn	µg·g^-1^	84.0 (0.71) a	57.0 (5.12) b	25 - 60
Mn		217.0 (2.42) b	369.3 (19.23) a	55 - 110
Fe		141.8 (10.28) a	136.3 (15.77) a	50 - 150
Cu		8.3 (0.25) a	6.3 (0.48) b	6 - 16
B		42.5 (1.19) b	211.0 (10.29) a	15 - 45
Al		13.0 (2.16) a	7.8 (0.95) a	1 - 300

^1^Plant Analysis Handbook IV edition (2014), Eds: Bryson and Mills, Micro-Macro Publishing, Athens, GA, USA.

Data is shown on a unit dry weight basis. Least square means with different letters are statistically different (*P* < 0.05) between the fertilizer treatments. The standard error of the mean is shown in parentheses.

### 3.2 Experiment II

#### 3.2.1 Effect of high-dose organic mix on shoot biomass and tissue nitrogen content

**Figure 5:**
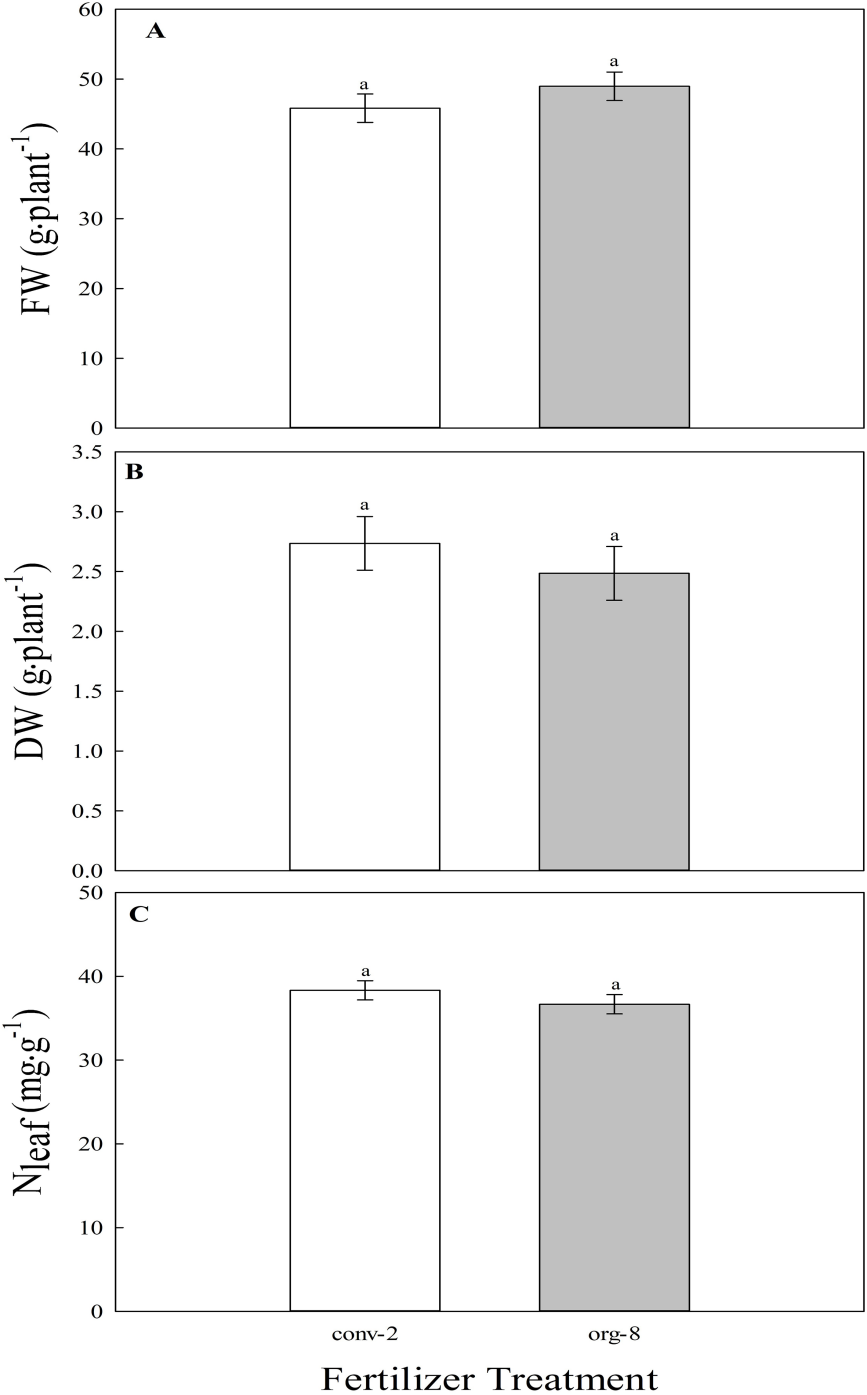
Main effect of fertilizer treatment [conventional (conv-2) and high-dose organic (org-8) mixes] on **(A)**. fresh weight (FW; g. plant^-1^), **(B)**. dry weight (DW; g. plant^-1^), and C. leaf nitrogen content (N_leaf_, mg·g^-1^) of lettuce in the first trial of experiment II. Bars represent the average of two cultivars (‘Waldmann’s dark green’ and ‘Red sails’) and four replications (N = 8). The least-square means with different letters are statistically different (*P* < 0.05). Error bars indicate the standard error of the mean.

There were no statistical differences between the conventional and high-dose organic mixes for FW and DW (**Figures 5A, B**) of lettuce in trial 1. Averaged across both cultivars, the FW of lettuce supplied with high-dose of organic mix was 48.97 g·plant^-^¹, which was statistically not different from that supplied with conventional mix (45.84 g·plant^-^¹). Similar results were observed for DW of plants. There were no statistical differences between the two fertilizer treatments for N_leaf_ in trial 1 (**Figure 5C**). Lettuce grown in conventional and high-dose organic mixes had N_leaf_ levels of 38.3 and 36.7 mg·g^-1^, respectively.

#### 3.2.2 Effect of residual fertilizer in the substrate on shoot biomass

**Figure 6:**
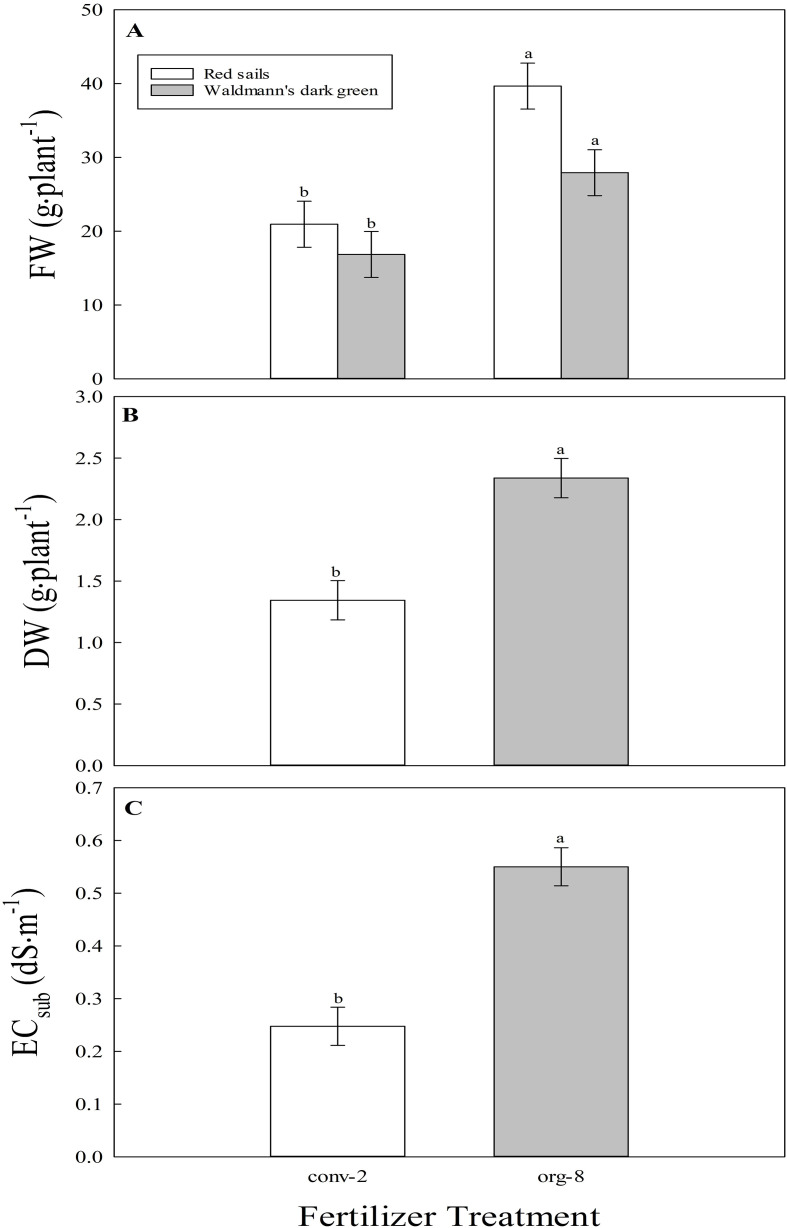
**(A)**. Interactive effect of fertilizer treatment [conventional (conv-2) and high-dose organic (org-8) mixes] and cultivar (‘Red sails’ and ‘Waldmann’s dark green’) on fresh weight (FW, g.plant^-1^) and main effects of fertilizer treatment on **(B)**. dry weight (DW, g.plant^-1^) and **(C)**. substrate electrical conductivity (EC_sub_, dS·m^-1^) of lettuce plants grown with residual fertilizer in the second trial of experiment II. Each bar represents the average of two varieties and four replications (N = 8). Least square means with different letters are statistically different (*P ≤* 0.05) between the fertilizer treatments. Error bars represent the standard error of the mean.

There was an interactive effect of fertilizer type (conventional and high-dose organic mixes) and cultivar (two cultivars) on FW of lettuce plants grown with residual fertilizer in the substrate (**Figure 6A**). The FW difference between the two fertilizer treatments was larger in ‘Red sails’ compared to ‘Waldmann’s dark green’. In ‘Red sails’ and ‘Waldmann’s dark green’ cultivars, the FW of plants grown with residual fertilizer was higher in the high-dose organic than conventional mix by approximately 100 and 45%, respectively. A main effect of fertilizer type was significant for DW and EC_sub_ when plants were grown with residual fertilizer (**Figures 6B, C**). Averaged across both cultivars, the DW of plants grown with residual fertilizer was approximately twice higher in the high-dose organic than conventional mix. A significantly higher EC_sub_ was observed at harvest stage in high-dose organic (0.54 dS·m^-1^) than conventional (0.24 dS·m^-1^) fertilizer treatment, with residual fertilizers.

## 4 Discussion

Unlike conventional fertilizers, organic fertilizers that can provide both macro and micro nutrients are unavailable. Moreover, available OMRI-certified organic fertilizers do not provide more than three plant nutrients in one compound. Given this, it is difficult to customize organic and conventional mixes with identical amounts of different nutrients. In our study, we prepared a customized organic mix that can supply most of the nutrients required for plant growth. The identified slow-release conventional fertilizer mix (Osmocote 5-9-12) contained most of the elements and was closest in composition to the customized organic mix after addition of gypsum. The quantity of nutrient elements in the conventional and organic mixes in experiment I was almost similar except for lower levels of P_2_O_5_ and S and higher levels of Fe and B in the organic compared to the conventional fertilizer (**Table 3**). However, these differences did not result in any observable nutrient deficiency or toxicity symptoms in plants. Elements including Cu and Zn were absent in the organic fertilizer mix, but their levels were within a sufficiency range (**Table 4**) in the plant tissue. This is likely due to the presence of these elements as impurities in the fertilizer components used to prepare the mix.

Several lettuce cultivars recorded lower FW or economic yield in the organic mix than conventional treatment in our study (**Figure 1**). These results indicate that the organic fertilizer mix used in our study can reduce lettuce yields in hydroponic production. Further, the average reduction in yield loss can be significant (36%) with our customized organic fertilizer mix in hydroponic crop production. In support, a meta-analysis of field studies indicated that organically grown crop yields were lower by approximately 25% than those of conventionally grown crops (de Ponti et al., 2012; Seufert et al., 2012). Lower yield in organic systems was related to the limited nutrient availability to plants from organic compared to conventional fertilizers (Asadu et al., 2024). Although the organic sources are beneficial for building soil health by adding soil organic matter and improving microbial activity, they are slow at releasing nutrients (Hernández et al., 2016). The slow release of nutrients can potentially cause a mismatch between nutrient demand and supply. This may be the reason for lower yields in organic compared to conventional production systems.

The linear relation between ‘yield gap’ in the organic mix and FW of cultivars in the conventional mix (**Figure 2**) in our study suggest that production losses can be higher in cultivars with higher growth rate than those with slower growth rate. It is likely that the nutrient demand is more in cultivars with fast than slow growth rate, leading to observed larger yield gap. The large size cultivars likely benefited from the readily available nutrients in the conventional than organic mix. Further, slow nutrient release in the organic mix had a relatively smaller negative effect on growth in red than green cultivars. In general, red leaf cultivars of lettuce have slower growth rate than green leaf cultivars (Miller et al., 2020), which was also observed in our study except for ‘Red sails’ cultivar (**Figure 1**). The red color of leaves in these cultivars is due to increased biosynthesis of anthocyanin pigment which competes with growth for photosynthates (Son and Oh, 2013; Kong and Nemali, 2023). Due to a relatively slower growth rate, it is likely that the demand for nutrients was relatively lower in red than green cultivars, thereby a smaller negative effect on growth. These results support that organic fertilizer management can be relatively more important when fast growing cultivars are used in crop production.

A higher EC_sub_ in the conventional than organic mix (**Figure 3**) indicates an increased level of nutrients available in the root zone of conventionalmix, even when both fertilizer treatments contained a similar quantity of nutrients. This further supports that lower availability of nutrients was the likely reason for lettuce plants exhibiting reduced biomass production in the organic mix used in our study. Organic fertilizers are prepared from plant and animal materials such as manures, blood or bone meal, and fish emulsions (Nygaard Sørensen and Thorup-Kristensen, 2011; Panday et al., 2024; Ullah et al., 2023). These fertilizers are complex forms that require microbes for decomposition into simpler forms. Mineralization converts organic nutrients into inorganic forms for root absorption (Paul, 2015; Harraq et al., 2022). The whole process of nutrient conversion takes time and often fails to keep pace with the plant nutrient demand (Evanylo et al., 2008; Nair et al., 2014). In addition, microbial activities depend on environmental conditions such as moderate soil temperature, adequate moisture, and a neutral to slightly acidic environment (Chapin, 2011; Bian et al., 2022; Wang et al., 2024).

The EC_sub_ patterns during 1, 10, and 20 DAT (**Figure 4**) suggest differences in both nutrient release and nutrient uptake by plants in the organic and conventional mixes. The day 10 after transplanting is closer to the end of lag (slow growth) and beginning of log (rapid growth) phase in lettuce (Li et al., 2020). The plant growth and nutrient uptake rates were likely slower during the lag phase (i.e., between 1 and 10 DAT). Therefore, the nutrients released from fertilizers in both treatments likely accumulated in the substrate. This likely led toan increase in EC_sub,_ albeit at a lower level in the organic mix. The higher EC_sub_ in conventional than in organic mix during this stage indicates that nutrients were released more rapidly to the substrate in the conventional mix. The higher EC_sub_ in the conventional than in the organic mix at the start of the log phase likely supported nutrient demand from plants and increased plant growth during this stage. A faster growth rate further likely increased nutrient uptake, which in turn led to an increased depletion of nutrients and EC_sub_ by 20 DAT. On the other hand, EC_sub_ in the organic mix showed a gradual decline between 10 and 20 DAT, which is likely due to a relatively slower growth rate from a lower level of EC_sub_. This likely reduced nutrient availability and nutrient uptake by plants during the log phase, leading to reduced growth in this treatment. Therefore, the differences in biomass production between conventional and organic treatments in our study are likely due to temporal changes in the availability of nutrients in the substrate, leading to a mismatch between nutrient demand by plants and nutrient supply by the organic compared to conventional mixes used in our study.

Although the levels of several elements (S, Zn, Cu, K, Mn, and B) were significantly different between the two fertilizer treatments, most of the levels were within the sufficiency range described for lettuce (Bryson et al., 2014). The organic mix contained a lower level of S and higher level of B than conventional mix (**Table 3**), which resulted in a lower S and higher B levels in the plant tissue in this treatment albeit being in the sufficiency range. Moreover, there were no toxicity symptoms from supra-optimal levels of Mn and B observed in plants. Despite Ca being deficient in both treatments, there were no deficiency symptoms (e.g., leaf tip burn) in plants. Although not statistically different between the two fertilizer treatments, Mg was deficient in the organic mix. However, the plants in the organic mix in our study did not exhibit any Mg deficiency symptoms (e.g., interveinal chlorosis). Therefore, it is unlikely these elements caused the observed differences in FW and DW between the fertilizer treatments.

Among different elements, N was the only element that was significantly lower and within deficiency range in plants subjected to the organic mix (**Table 4**). The plants in the organic mix in our study did exhibit symptoms of N deficiency (e.g., reduced vegetative growth). These results indicate that N deficiency was the major factor responsible for observed biomass differences between the two fertilizer treatments in our study. It is well known that N is a major component of chlorophyll molecule responsible for light absorption (Evans and Terashima, 1987) and constituent of rubisco enzyme involved in carboxylation (Makino, 2003). Plants preferentially lower shoot growth to promote root growth for increased absorption in response to N deficiency (Evans and Poorter, 2001). Another symptom of N deficiency is a decrease in leaf growth (Adhikari and Nemali, 2023), which further lowers light interception by plants. Therefore, we hypothesize that the lower biomass production in the organic compared to conventional mix in our study is due to slow nutrient release from the organic mix, leading to N deficiency and low levels of N_leaf_. These differences likely affected photosynthetic performance and leaf growth in plants subjected organic treatment.

Experiment II was designed to test the hypothesis from experiment I and to identify a fertilizer management strategy to minimize the yield gap between conventional and organic mixes. No significant differences in FW and DW between conventional and high-dose organic mixes (**Figures 5A, B**) suggest that increasing the amount of customized organic mix incorporated into the substrate can increase nutrient availability and biomass production. In support, de Ponti et al. (2012) found that organic productivity relies on organic nutrient management, and abundant application of organic fertilizer increased the yield of field-grown organic crops. Although not statistically different, observed trends in FW and DW were not similar between the two treatments (**Figure 5**). Specifically, FW was slightly lower while DW was slightly higher in the high-dose organic than conventional mix. Slightly lower FW in the high-dose organic treatment could be due to differences in plant water status (as FW is mostly comprised of water weight) between conventional and high-dose organic mixes. It is possible that a high dose of organic mix in the substrate mildly increased osmotic stress and reduced the overall water uptake by plants. This may have resulted in a numerically lower FW in the high-dose organic than conventional mix. Further, no differences in yield were associated with lack of differences in N_leaf_ between conventional and high-dose organic mixes (**Figure 5C**), indicating that a higher dose of organic mix can enhance availability and uptake of N. These results support our hypothesis that low N_leaf_ was the predominant physiological trait influencing biomass production in organic mix used in our study.

A significantly higher EC_sub_ in the high-dose organic mix than in the conventional mix with residual fertilizer (**Figure 6C**) indicates that more nutrients were likely left over in the substrate with high-dose organic mix. Therefore, it is likely that not all of the substrate-incorporated organic fertilizer in the high-dose mix was completely utilized by plants in trial 1. To support this, the average FW of lettuce with residual fertilizer in the high-dose organic mix was higher than that of conventional mix (**Figure 6A**). Moreover, plants were of good size (approximately 69% of the average FW of the cultivars in trial 1) in the high-dose organic mix. Interestingly, the combined FW of lettuce plants from both trials was higher in the high-dose organic (82.8 g·pot^-1^) than in the conventional (64.7 g·pot^-1^) mix.

Based on our results, a high-dose of organic mix (similar to the one used in our study) can be used to potentially increase lettuce yield. This is only when the substrate is reused at least in two crop cycles. This approach will likely result in lowering costs and increasing yields and nutrient use efficiency in organic systems. Moreover, reusing the substrate with residual fertilizer can reduce environmental pollution. Precipitation can cause nutrients in the discarded substrate to leach and run off into natural water bodies. However, there are some considerations for reusing the substrate in subsequent production cycles, including: (i) necessity to add fresh substrate material in the second cycle to compensate for loss of substrate from cleaning and removing plant debris/roots, (ii) composition of residual nutrients in the substrate will likely be different from the original mix due to differential nutrient uptake rates by plants (Bugbee, 2004). This may lead to a deficiency of specific nutrients, especially those that are rapidly absorbed by plants, in the second cycle. (iii) reused substrate can harbor pathogens or accumulate phytotoxic substances, which may affect crop growth. However, we did not observe any deficiency or toxicity symptoms using our customized organic mix. Additional research will be needed to thoroughly validate the possibility of reusing residual nutrients and substrate hydroponic production. In addition, certain organic materials (e.g., biochar, Zabaleta Nievas et al., 2023), when incorporated into organic substrates can increase nutrient supply. The potential use of such materials to increase nutrient release from organic mixes should be tested in future studies.

## 5 Conclusions

Our research indicates that yield loss in hydroponically-grown lettuce can be significant when using a customized organic fertilizer mix developed in our study. This was due to slower nutrient release in the customized organic than conventional mix. The lower nutrient availability in the substrate resulted in reduced nutrient uptake and N deficiency in plants subjected to organic mix. A lower N_leaf_ was responsible for reduced biomass production in the organic compared to conventional fertilizer mix. Further, our results suggest that organic fertilizer management can be more important when fast-growing lettuce cultivars are used in hydroponic production due to their higher nutrient demand. Although a high-dose of organic mix improved nutrient availability, uptake, and increased biomass production, the amount of residual nutrients in the substrate with high-dose organic mixes can be significant. Therefore, a high-dose organic fertilization strategy can only be practical when the substrate is reused in subsequent cycles. Future research should focus on strategies that enhance nutrient release from organic fertilizers, such as using organic amendments or microbial supplements.

## Data Availability

The raw data supporting the conclusions of this article will be made available by the authors, without undue reservation.
